# Deep Learning-Based Vehicle Classification for Low Quality Images

**DOI:** 10.3390/s22134740

**Published:** 2022-06-23

**Authors:** Sumeyra Tas, Ozgen Sari, Yaser Dalveren, Senol Pazar, Ali Kara, Mohammad Derawi

**Affiliations:** 1 Graduate School of Natural and Applied Sciences, Atilim University, Incek Golbasi, Ankara 06830, Turkey; smeyratas@gmail.com (S.T.); ozgensari@hotmail.com (O.S.); 2 Department of Avionics, Atilim University, Kizilcasar Mahallesi, Incek Golbasi, Ankara 06830, Turkey; yaser.dalveren@atilim.edu.tr; 3 Department of Computer Programming, Biruni University, Istanbul 34010, Turkey; spazar@biruni.edu.tr or; 4 Ankageo Co. Ltd., Yildiz Technical University Ikitelli Technopark, Istanbul 34220, Turkey; 5 Department of Electrical and Electronics Engineering, Gazi University, Eti Mahallesi, Yukselis Sokak, Maltepe, Ankara 06570, Turkey; akara@gazi.edu.tr; 6 Department of Electronic Systems, Norwegian University of Science and Technology, 2815 Gjøvik, Norway

**Keywords:** vehicle classification, convolutional neural network, deep learning, low resolution, low quality

## Abstract

This study proposes a simple convolutional neural network (CNN)-based model for vehicle classification in low resolution surveillance images collected by a standard security camera installed distant from a traffic scene. In order to evaluate its effectiveness, the proposed model is tested on a new dataset containing tiny (100 × 100 pixels) and low resolution (96 dpi) vehicle images. The proposed model is then compared with well-known VGG16-based CNN models in terms of accuracy and complexity. Results indicate that although the well-known models provide higher accuracy, the proposed method offers an acceptable accuracy (92.9%) as well as a simple and lightweight solution for vehicle classification in low quality images. Thus, it is believed that this study might provide useful perception and understanding for further research on the use of standard low-cost cameras to enhance the ability of the intelligent systems such as intelligent transportation system applications.

## 1. Introduction

The classification of road vehicles is one of the important challenges in the Intelligent Transportation System (ITS) applications such as roadway tolling, road surveillance/planning, traffic safety, autonomous driving, and parking lot management systems [1,2,3]. Over the years, numerous studies have been proposed in the literature to ease this challenge. Mainly, vehicle classification systems can be grouped into two categories, namely sensor-based methods [4,5,6,7,8] and vison-based methods [9]. In sensor-based methods, typically, the different types of sensors such as magnetic sensors [4,5], microwave radar sensors [6], and Anisotropic Magnetoresistive Sensors (AMR) [7,8] are used to classify the vehicles. However, the methods based on these sensor technologies have some limitations and difficulties regarding costs, deployment, and accuracy. On the other hand, vision-based methods rely on the use of image sequences of traffic scenes obtained by a camera [9]. Vison-based systems provide several advantages when compared with sensor-based methods. One of the important advantages is related to the ease of camera installation, which significantly reduces the cost and difficulties in the system design, deployment, and maintenance. Moreover, reliable data collected by the automated system can lead to more efficient classification performance.

In recent years, developing vision-based vehicle classification methods using machine learning (ML) has attracted many researchers, as it offers an efficient and adaptable approach that can fulfil the requirements of growing ITS applications. In this context, a large number of works that apply ML methods for vision-based vehicle classification have been proposed so far [10,11,12,13,14,15,16,17,18,19,20,21,22,23,24,25]. In [10], a method for vehicle image classification using neural network (NN) with conditional adaptive distance is presented. The vehicle classification method based on the use of multi-class support vector machine (SVM) is proposed in [11]. In [12], another a vehicle classification method that adopts fuzzy support vector machine is provided. In [13], AdaBoost method is used for vehicle classification. Another classification method using semisupervised convolutional neural network (CNN) is presented in [14]. In [15], a progressive CNN architecture is used for vehicle classification. Similarly, a CNN-based vehicle type classification system is proposed in [16]. Moreover, a vehicle classification from the CNN pre-trained dataset is presented in [17]. Additionally, a simple algorithm in which a deep CNN model is represented for vehicle classification is provided in [18]. In [19], a framework for vehicle classification using deep learning neural network (Inception-v3 model) is presented. Another study that uses CNN for vehicle classification is proposed in [20]. Furthermore, a real-time vehicle type classification system based on Faster Region-convolutional neural networks (Faster R-CNN) is presented in [21]. Apart from this, an improved Faster R-CNN method-based vehicle classification is also presented in [22]. In [23], a vehicle classification and counting method based on CNN models is proposed. In [24], vehicle classification by stacking ensemble of three deep neural networks is presented. A deep learning-based object detection algorithm (SSD: Single Shot MultiBox Detector) is also proposed for vehicle classification in [25]. It is important to note that although various classifiers have been used in these works, CNN retains its popularity due to its superior performance on the large-scale image datasets.

In ITS applications, surveillance or monitoring cameras, which provide high quality videos/images in terms of frame rate or resolution, are mostly preferred for real-time monitoring. The camera view is set to the region of interest (ROI) such as a traffic scene, a road, or a highway. However, the system cost is one of the major concerns in the deployment of ITS applications. On the other hand, apart from high level ITS applications, typical low-cost traffic surveillance cameras can be employed in low-cost traffic monitoring systems, as they are mostly used in third world countries. Nevertheless, this type of cameras usually provides lower image resolution quality, which leads to less pattern information because of low signal-to-noise ratio (SNR). Particularly, when the images of distant vehicles are concerned, the classification becomes quite difficult, as such types of images are tiny and often low resolution.

### 1.1. Related Work

In order to deal with the aforementioned challenges, researchers have started to investigate the implementation of deep learning approaches based on CNN in recent years [26,27,28]. In [26], a CNN-based vehicle detection and classification system using a low quality real-time monitoring camera is proposed. To evaluate the applicability of CNN in real-time applications, detection and classification execution time are comparatively assessed by using both the CPU and GPU. The study presented in [27] proposes another method based on Faster R-CNN architecture to detect and classify the distant vehicles in real-time applications. The performance of the design proposed in the study is assessed under different weather conditions. Moreover, in [28], the problem of low resolution in classifying tiny objects is investigated. To solve this problem, a method that employs generative adversarial network (GAN) with two CNNs is proposed. In the proposed method, high resolution images from low resolution images are generated to provide more correct images for the classifier.

As can be deduced from the brief discussion above, only a few studies exist on the development of deep learning-based vehicle classification methods for low quality images. It is worth noting that the data used in these studies are collected by monitoring cameras with a depression angle view and/or dashcam view, where the camera view is set to ROI. Additionally, in the studies, the cameras are not distant enough from the ROI. In this context, to the best of the authors’ knowledge, there has been no published work regarding deep learning-based vehicle classification for low quality images collected by a low resolution surveillance camera with a wide angle view, which is installed distant from the ROI and used for security purposes rather than traffic monitoring.

### 1.2. Contributions

This article is devoted to address a concern regarding how standard cameras, which are deployed in any place and used for different purposes rather than traffic monitoring, can be utilized to increase the capabilities of intelligent systems such as ITS applications. In this context, the main idea is to extract meaningful insights from the recordings of a particular location. Thus, as a case study, we aimed at developing a simple but accurate CNN-based model for vehicle classification in low resolution surveillance images (96 dpi) collected by a standard security camera installed distant from the ROI. To this end, firstly, a dataset containing low resolution vehicle images (4800 images) cropped from the surveillance video frames is created [29]. In order to classify the vehicles, a CNN-based model built from scratch is proposed. The performance of the proposed model is then compared with the well-known and efficient models such as the VGG16 pre-trained model and the VGG16 pre-trained fine-tuning model in terms of the accuracy and complexity. From the comparison results, it is reported that the VGG16 fine-tuning model provides higher accuracy (99.2%) in vehicle classification for low quality images. However, although the proposed model provides an acceptable accuracy (92.9%), it is simple and lightweight due to the lesser number of layers (nine layers) and parameters (around 17 k) used in its architecture. Moreover, the proposed model provides faster training time (6 min). These advantages make the proposed model as energy efficient as the other well-known VGG16 models in practice. Therefore, in a broad sense, it is shown that vehicle classification is possible even with a small dataset containing low resolution surveillance images collected by a standard surveillance camera. Additionally, the proposed model is a good candidate for the classification of vehicles with low quality images in terms of size and resolution.

As a summary, the main contributions of this study are listed as follows:

(a) A new dataset containing tiny and low quality vehicle images collected by a standard security camera, which is installed distant from the ROI, is created (imperfections on the camera and its installation are introduced together as per typical ITS application).
(b) A novel CNN model is developed for the classification of low quality vehicle images, and its accuracy is compared with well-known CNN models.
(c) The proposed model is shown to achieve an acceptable accuracy with its lightweight solution even if a small dataset containing low resolution surveillance images is used.

The rest of the paper is organized as follows. In the following section, the proposed model and other models used for the performance comparison are presented. In Section 3, experiments carried out within the context of the study are described. Experimental results and discussions are provided in Section 4. Finally, the paper concludes in Section 5.

## 2. Models for Classifying Low Quality Vehicle Images

### 2.1. The Proposed Model

**Figure 1:**
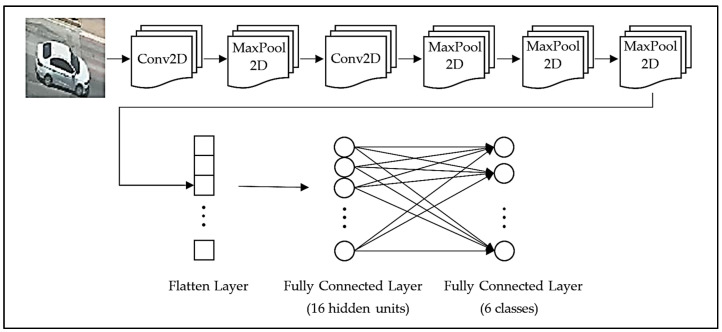
Architecture of the proposed model.

The architecture of the proposed model is shown in Figure 1. As shown in the figure, in the first stage of the architecture, which corresponds to the feature extraction network, two convolutional layers (Conv2D) and four max pooling layers (MaxPool2D) are used. Each of the Conv2d layers has 16 filters with 5 × 5 filter size, and both layers utilize Rectifier Linear Unit (ReLU) as the activation function. Each of the MaxPool2D layers, on the other hand, has 2 × 2 filter size and a stride value of 2.

In the second stage of the architecture, there is a flatten operation, which is applied to convert the feature map into a column vector. This is followed by a fully connected layer consisting of 16 hidden units, where a L2 regularizer is applied at a rate of 0.008 (to prevent overfitting) and ReLU is utilized as the activation function. Then, a dropout layer is used to randomly drop out the nodes, where the dropout rate is set to be 0.3 (30%). It should be noted that the main motivation to select the parameters both in fully connected layer and dropout layer is to prevent the model from overfitting. In the last stage of the architecture, there is a final fully connected layer that consists of six nodes (classes) for classification using Softmax activation.

### 2.2. VGG16 Pre-Trained Model

The VGG16 is a well-known CNN architecture and widely used in many deep learning image classification techniques [30]. Due to its ease of implementation, the VGG16 retains its popularity in learning applications. Basically, a VGG16 network is trained on a dataset called as ImageNet, which contains more than 14 million images. It is then obvious that the use of this pre-trained network could be an efficient means to improve the accuracy of the proposed model.

**Figure 2:**
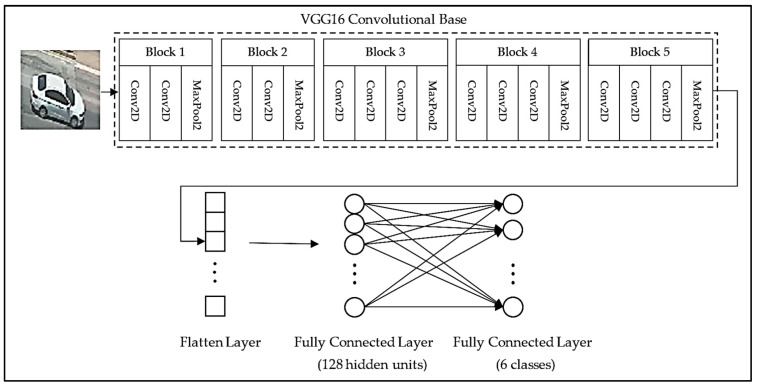
Architecture of the VGG16 pre-trained model.

In the first stage of the model architecture, a convolutional base of the VGG16 network consisting of five blocks, each of which has own convolutional and max pooling layers, is used as shown in Figure 2. Similar to the proposed model, the remaining stage of the architecture consists of the flatten layer, a fully connected layer, a dropout layer, and a final fully connected layer. Here, the only difference is that there are 128 hidden units in the fully connected layer. It is important to note that the convolutional base is frozen during the training process so that the pre-trained weights could remain unaltered.

### 2.3. VGG16 Fine-Tuning Pre-Trained Model

Fine-tuning is a method that is used to unfreeze a few of the top layers of a frozen model base. In general, it jointly trains both the last layers of the base model and the added classifier layers. In this way, the feature representations in the base model become more appropriate for a given specific task. This suggests that it might be possible to achieve better accuracy by applying the fine-tuning method to VGG16 pre-trained model. Thus, in this model, fine-tuning is applied to the convolutional base of the pre-trained model shown in Figure 2. To this end, the convolution layers in the last block of the convolutional base are unfrozen during the training process.

## 3. Experiments

### 3.1. Dataset and Preprocessing

**Figure 3:**
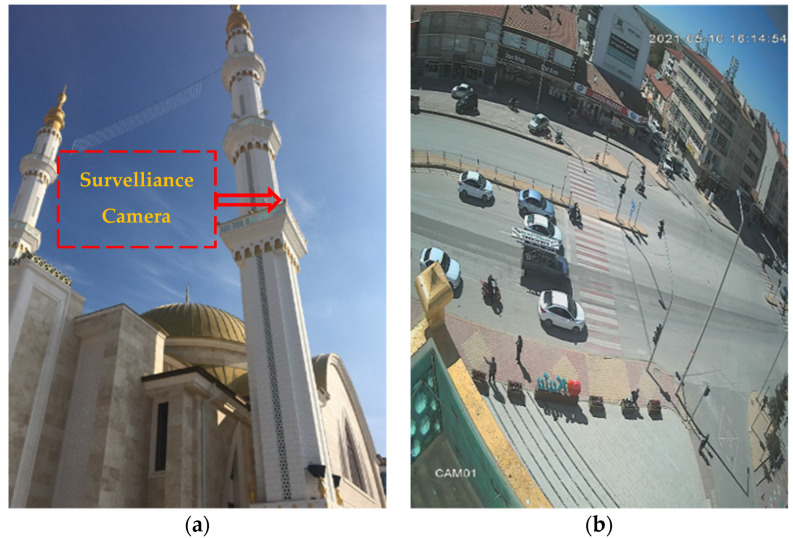
(**a**) Position of the camera placed on the minaret and (**b**) a view from the camera.

In this study, a new dataset containing low quality vehicle images was created [29]. To do this, firstly, we gathered a set of video recordings captured by a standard surveillance camera monitoring a particular square in Konya city, Turkey, for security purposes. Figure 3a shows the position of the camera, which was placed on one of the minarets of a mosque located in Konya. From Figure 3b, it can be easily seen that the camera has a wide view, and it is distant from the traffic scene, which is considered as the ROI in this study.

**Figure 4:**
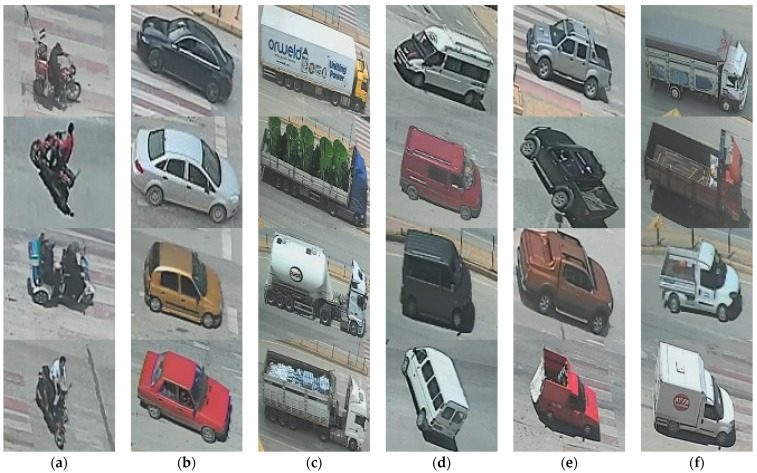
Samples of vehicles: (**a**) bike, (**b**) car, (**c**) juggernaut, (**d**) minibus, (**e**) pickup, and (**f**) truck.

After gathering the recordings, the ROI was zoomed-in to obtain more visible, clear images to be used in the network. Then, the images with 96 dpi resolution were cropped from the zoomed video frames. The vehicles in the images were grouped into six classes: bike, car, juggernaut, minibus, pickup, and truck. For each class, 800 vehicle images were collected. Thus, a dataset containing 4800 vehicle images was created. As an example, Figure 4 shows the different samples of vehicles after manually cropping the images from the video frames.

**Figure 5:**
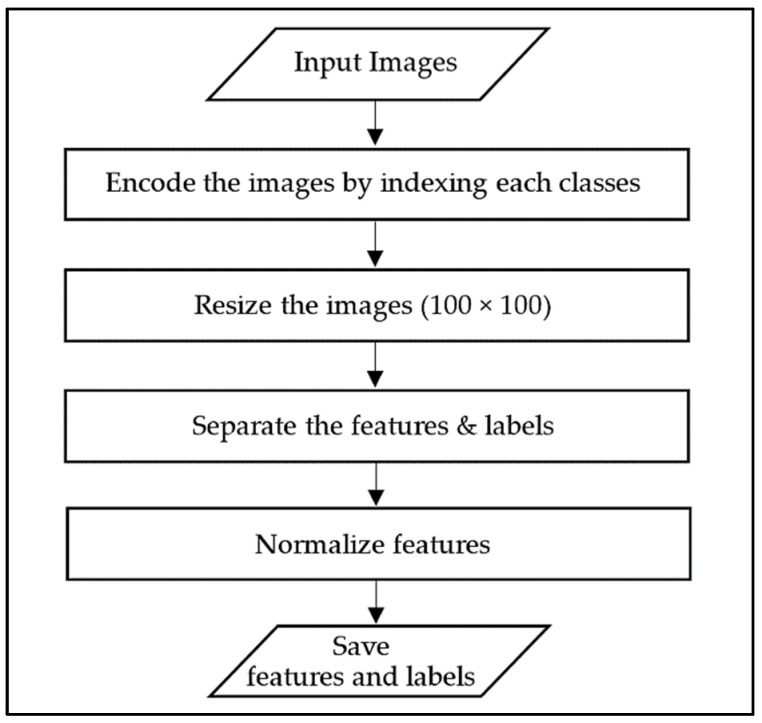
The flowchart of data preprocessing.

The next step after determining the classes, the data were preprocessed before it was fed into the networks during training. The flowchart representing the stages involved in the data preprocessing is shown in Figure 5. As shown in the figure, firstly, the data were encoded by indexing each class. All data were then resized to 100 × 100 pixels. Next, the features and labels were separated from each other, followed by the feature normalization.

### 3.2. Parameters and Training Details

Before the experiments, the dataset prepared for the network was separated into the train, validation, and test set. Here, the train set was used to train the model whereas the validation and test sets were used to evaluate model forecasting performance on never-seen data. More precisely, the validation set was used to tune the network hyperparameters except for parameters and learnable values (weights and biases), while the test set was used to see how the trained model could generalize its results on other new data. In the experiments, both the test set and validation set consisted of 480 vehicle images (10% for both), while the train set consisted of the remaining 3840 vehicle images (80%).

In order to train the models, RMSprop optimizer was used. This is because more stable training performance was achieved in initial experiments when compared to SGD and Adam optimizers. The training of the proposed model was completed in 40 epochs where the batch size was 32 and the learning rate of the optimizer was set to 0.001. On the other hand, the training of other models was completed in 25 epochs where the batch size was 32 and the learning rate of the optimizer was set to 0.0001. The metric used was accuracy, and sparse categorical cross-entropy was used to calculate the validation loss.

The algorithms were realized with Python 3.8 using TensorFlow, Keras, and Sklearn libraries. All networks were trained and tested on a PC server, the specifications of which are listed in Table 1.

### 3.3. Results

**Figure 6:**
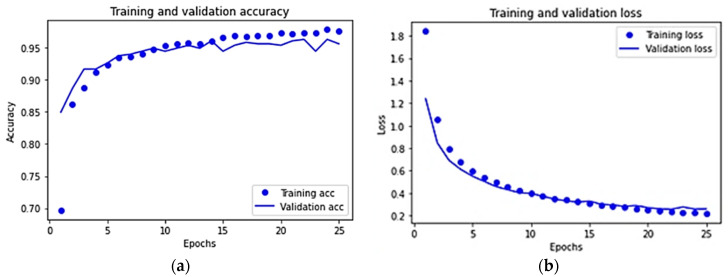
For the proposed model: (**a**) training and validation accuracy, and (**b**) training and validation loss.

The CNN-based models presented in Section 2 were tested on the created dataset in order to assess their effectiveness. Figure 6a shows the training and validation accuracy, and Figure 6b shows the training and validation loss of the proposed model. The diagrams indicate that there is no overfitting problem and the test accuracy is acceptable, where the accuracy is found to be 92.9% and the loss is found to be 30.3%.

**Figure 7:**
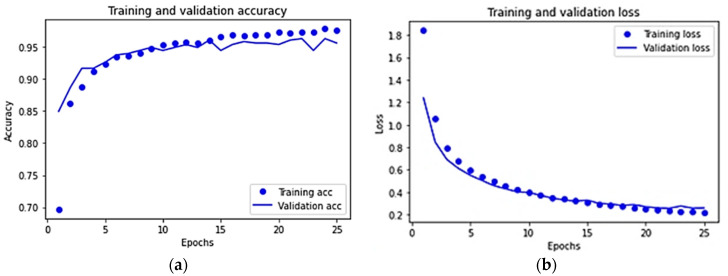
For the VGG16 pre-trained model: (**a**) training and validation accuracy, and (**b**) training and validation loss.

For the VGG16 pre-trained model, the training and validation accuracy and loss diagrams are shown in Figure 7a and Figure 7b, respectively. The diagrams show that the model is well-trained and overfitting is not observed in the results. In comparison to the proposed model, the improved accuracy and reduced loss with successive epochs can also be observed for the VGG16 pre-trained model, where the maximum accuracy is found to be 96% while the minimum loss is found to be 24.7%.

**Figure 8:**
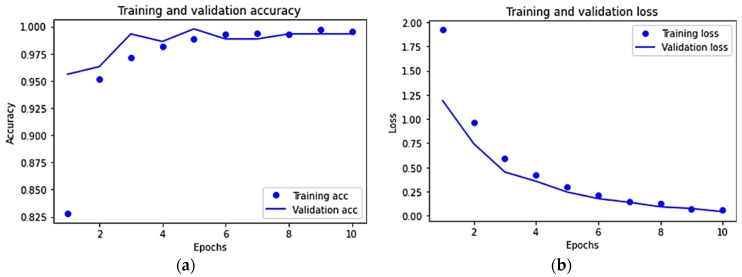
For the VGG16 fine-tuning pre-trained model: (**a**) training and validation accuracy, and (**b**) Training and validation loss.

On the other hand, for the VGG16 fine-tuning pre-trained model, the training and validation accuracy and loss diagrams are shown in Figure 8a and Figure 8b, respectively. The improvement on the accuracy and the reduction on the loss with successive epochs can be clearly observed from the results. More specifically, when compared to other models, the VGG16 fine-tuning pre-trained model achieves higher test accuracy with 99.2% while it achieves smaller loss found to be 7.7%.

Table 2 shows the comparative performances of the CNN-based models. Obviously, the VGG16 fine-tuning pre-trained model demonstrated the highest accuracy. It is followed by the VGG16 pre-trained model, which performed as the second most efficient network with an accuracy of 96%. With an accuracy of 92.9%, the performance of the proposed model was the least efficient network. However, it is important to note that there is a trade-off that needs to be taken into account, which is the complexity of the models versus their accuracies. It is already known that the design space is increased when the number of parameters of a CNN model is increased [31]. In this case, the number of design points is also increased, which provides more efficient solution, and hence, the learning process of the network is simplified. In practice, however, when the energy consumption and hardware limitations are concerned, the complexity reduction is certainly required at the expense of accuracy degradation. This can be clearly observed from the results listed in Table 2. Apparently, although the proposed method seems to be less efficient, it is fast, simple, and lightweight when compared to other models in terms of the training time, the number of layers, and the parameters.

Furthermore, the emerging era of big data has resulted in complex data that requires fast and effective decision making. The small datasets, however, lead to difficulties in decision making and data analysis. Therefore, the use of small datasets is mostly avoided, as it is inadequate to build an efficient prediction model. In this context, as discussed in [32], it is difficult to achieve higher accuracy rates (typically, over 85%) by a model built from scratch with a small dataset. However, the results listed in Table 2 show that the acceptable accuracy can be achieved by the proposed model (92.9%) even when a smaller dataset (containing 4800 images) is used.

Overall, the results achieved from the experiments prove that the accuracy of the proposed model is acceptable even when a small dataset is used, and it could be a simple and lightweight alternative for the classification of low quality vehicle images. The results also suggest that it is possible to classify vehicles in low resolution surveillance images collected by a standard security camera installed distant from the ROI.

## 4. Further Discussions and Future Work

The use of a robust vehicle detection method has an important role in a traffic monitoring system to provide an efficient vehicle classification. In this study, an automated vehicle detection method was not used, due to the fact that the main efforts are concentrated on the classification of low quality vehicle images in a simple but accurate way. Instead, the vehicle images were manually cropped from the video frames, and then stored in a dataset. Therefore, the detection of the vehicles could be an open issue that might be resolved by integrating an effective vehicle detection algorithm to the presented classification schemes.

On the other hand, as is known, the datasets are very important for solving object classification problems by using machine learning. For this reason, several open source datasets have been presented to assist many researchers working on the development of vision-based classification methods [33,34]. However, these datasets contain high quality images. For this reason, these datasets were not utilized to test the effectiveness of the models presented in this study. As an additional note, the dataset created in this study will be open for the research community in the near future.

Another open issue can be linked to the classification accuracy of the presented models under the common challenges of the vision-based classification systems that adversely affect their performance, such as various lighting conditions, different weather conditions, and image blurring. In order to address these challenges, the authors are currently working on the development of a simple deep learning-based model. In this context, a new dataset containing 4800 low quality vehicle images with 100 × 100 pixels and 96 dpi resolution under different weather conditions is expected to be created. To do this, the data collection system used in this study will be applied. Then, a simple CNN-based model to be an alternative to well-known CNN models in terms of short training time, the small number of layers along with the parameters, is expected to be developed.

It is also important to note that although the VGG16 might be considered as a relatively old pre-trained network, it is still widely used in the benchmarking of such model development, and researchers are more familiar with VGG16 [35]. On the other hand, in this work, our focus is not the performance of VGG16 but our simple model for such unstudied classification problems considering low quality vehicle images captured by imperfectly positioned standard cameras.

## 5. Conclusions

In this study, the purpose was to classify the vehicles in low resolution surveillance images which were collected by a standard camera installed distant from the ROI such as a traffic scene, a road, or a highway. To this end, a novel CNN model built from scratch along with well-known CNN models based on VGG16 were proposed, and their accuracy was evaluated on a new dataset containing tiny and low quality vehicle images. According to the test results, the VGG16 fine-tuning pre-trained model demonstrated the highest accuracy with 99.2%. This is followed by VGG16 pre-trained model, which performed with an accuracy of 96%. The proposed model, on the other hand, provided acceptable accuracy, which was found to be 92.9%. Apparently, results show the efficiency of the VGG16-based CNN models; however, the proposed method offers significant advantages over the VGG16 fine-tuning and VGG16 pre-trained models. One of them is that the proposed model is simple and lightweight due to the number of layers used in its architecture which consists of only 9 layers while the other models use 21 layers. Thus, at the expense of accuracy degradation, the complexity is reduced, where the number of parameters is around 17 k whereas the number of parameters used in other models is around 15.3 M. Therefore, as another significant advantage, the proposed method could be a reasonable option when the energy consumption and hardware limitations are concerned. Additionally, when compared to other models in terms of the training time, the proposed method seems to be fast enough. For the created dataset containing 4800 images, the elapsed training time of the proposed model was observed to be around 6 min while the training time of the VGG16 fine-tuning model and the VGG16 pre-trained model was around 15 min and 28 min, respectively. Moreover, results show that the proposed model provides acceptable accuracy without the need for a large dataset.

From a broader perspective, this study proves that vehicle classification is possible with low resolution surveillance images collected by a standard camera used for security purposes rather than traffic monitoring. In this context, we believe that the results achieved from this study will pave the way for further research on the use of standard security cameras to increase the capability of intelligent systems such as ITS applications.

## Figures and Tables

**Table 1:** The specifications of the server used in the study.

**CPU**	Intel Core i7-7500U @3.5 GHz
**GPU**	NVIDIA GeForce 920M
**Memory (RAM)**	8 GB
**Operating System**	Windows 10 (64 bits)

**Table 2:** Comparison of the test accuracy and loss for the CNN-based models.

CNN Models	Accuracy (%)	Loss (%)	# Layers	# Parameters	Training Time (Minutes)
Proposed Model	92.9	30.3	9	~17 k	~6
VGG16 Pre-trained Model	96	24.7	21	~15.3 M	~28
VGG16 Fine-tuning Pre-trained Model	99.2	7.7	21	~15.3 M	~15

## Data Availability

Data are available in a publicly accessible repository. The data presented in this study are openly available in Zenodo at https://doi.org/10.5281/zenodo.6634554, accessed on 22 May 2022.
